# ID 80 - Guia para Implementar um Núcleo de Evidências em Saúde (NEv)

**DOI:** 10.5327/2237-9622.2025.v34s1.80

**Published:** 2025-11-25

**Authors:** Rachel Riera, Davi Mamblona Marques Romão, Maria Lúcia Teixeira Machado, Jorge Otávio Maia Barreto, Cecília Setti, Romeu Gomes, Silvio Fernandes da Silva, Aurelina Aguiar de Lima, Daienne Amaral Machado, Fernanda Campos de Almeida Carrer, Maritsa Carla de Bortoli, Natan Monsores de Sá, Nathan Mendes Souza, Roberta Borges Silva, Sandra Maria do Valle Leone de Oliveira, Vahíd Shaikhzadeh Vahdat, Tereza Setsuko Toma

**Keywords:** Política Informada por Evidências, ensino, aprendizagem, Educação em Saúde, Gestão da Informação em Saúde

## Abstract

**Introdução:**

Núcleos de Evidências (NEv) representam importante estratégia para apoiar instituições e agentes políticos e sociais no uso de evidências na tomada de decisão. As reflexões necessárias no processo de criação e manutenção de um NEv são abordadas neste Guia.

**Método:**

O Guia foi elaborado por especialistas em políticas informadas por evidências (PIE). Uma série de atividades para coleta de insumos e validação de conteúdos foi desenvolvida previamente: um inventário de referências, um questionário e duas oficinas de trabalho com profissionais que atuam ou conhecem NEv. Em seguida, ocorreu o processo de autoria do Guia NEv com a realização de cinco oficinas de trabalho.

**Resultados:**

O NEv pode ser compreendido como um "intermediário de evidências" – uma unidade que facilita o acesso das pessoas envolvidas na formulação e na implementação de políticas públicas às evidências necessárias. Ele pode fazer parte de diferentes tipos de organizações (governos, universidades, terceiro setor ou setor privado). Os produtos e os serviços ofertados pelo NEv são caracterizados pela transparência, sistematicidade e participação social. É desejável que o NEv conte com, ao menos, os seguintes elementos constitutivos: equipe de, no mínimo, duas ou três pessoas, com qualificação adequada para os serviços que serão ofertados; espaço físico e/ou recursos para trabalho remoto; recursos de informática (computadores, acesso à internet, armazenamento e segurança da informação); acesso a recursos para busca de evidências (bancos de dados, repositórios de publicações etc.). A dinâmica de trabalho do NEv vai envolver a identificação de uma demanda de política, a definição de uma estratégia de tradução de conhecimento adequada para atender a essa demanda, a execução desta estratégia, a finalização do trabalho e entrega dos produtos acordados, a disseminação dos resultados e a avaliação do processo. A participação do NEv em redes de PIE gera muitas oportunidades de troca e trabalho em parceria, sendo uma das principais referências a EVIPNet (Rede para Políticas Informadas por Evidências). O Guia apresenta quatro passos para a criação de um NEv: Bases do NEv, descreve aspectos institucionais que devem ser trabalhados; Estrutura do NEv, aborda passos relevantes para a criação e manutenção da capacidade operacional do NEv, enquanto um serviço ou unidade organizacional; Gestão do NEv, discute aspectos importantes relacionados a sua gestão, de modo que o funcionamento seja profissional e técnico; Projeção do trabalho do NEv, apresenta passos que podem ser adotados para fortalecer a imagem e o reconhecimento do NEv.

**Conclusão:**

No mundo em constante transformação, os NEv devem manter um olhar atento às novas tendências, oportunidades e necessidades. A expansão de campos como a ciência aberta e ciência cidadã abrem novas possibilidades de produção de conhecimento sistemático e equitativo, representando diferentes grupos sociais. Os contínuos avanços em inteligência artificial e geração automática de texto devem paulatinamente automatizar algumas das tarefas técnicas do NEv. A equipe do núcleo precisará incorporar as novas ferramentas e aprender a usá-las para potencializar seu trabalho. Os NEv devem pensar estrategicamente como promover a cultura do uso de evidências, reconhecendo e incorporando as outras dimensões dos processos políticos.

